# A Case of Mitral Valve Infective Endocarditis and Atrial Fibrillation Complicated by Hemorrhagic Stroke: A Challenging Clinical Scenario and Approach to Management

**DOI:** 10.7759/cureus.41634

**Published:** 2023-07-10

**Authors:** Mubariz A Hassan, Niyati Grewal, Daniel Nepaul

**Affiliations:** 1 Internal Medicine, Howard University Hospital, Washington, D.C., USA; 2 Cardiovascular Disease, Howard University Hospital, Washington, D.C., USA

**Keywords:** therapeutic anticoagulation, acute hemorrhagic stroke, intravenous drug use (ivdu), atrial fibrillation (af), infective endocarditis

## Abstract

This case report presents a rare and intricate clinical scenario involving a 58-year-old male with a history of hypertension, intravenous drug use (IVDU), and cocaine abuse. The patient presented with profound hypotension and symptoms suggestive of impending shock. Septic workup revealed *Staphylococcus aureus* in all four blood culture bottles, confirming a diagnosis of infective endocarditis (IE). Transthoracic echocardiography demonstrated a large vegetation measuring 1.9x1.7 cm on the mitral valve. Additionally, the patient exhibited non-ST segment elevated myocardial infarction (NSTEMI) type II in the setting of cocaine use, atrial fibrillation, and therapeutic anticoagulation. Subsequent imaging studies raised concerns regarding hemorrhagic stroke. A multidisciplinary team comprising cardiology, cardiothoracic surgery, infectious disease, and neurology collaborated to develop an optimal management strategy. Considering the high-risk features of the IE and the need to address the hemorrhagic stroke, anticoagulation was temporarily halted, and the patient was transferred for urgent mitral valve replacement surgery. This case highlights the complex interplay between substance abuse, cardiovascular complications, IE, and neurological events, underscoring the challenges encountered in managing such patients.

## Introduction

Intravenous drug use (IVDU) is associated with a myriad of complications, including infective endocarditis (IE) and cardiovascular events [1]. Concurrent substance abuse, such as cocaine and heroin use, further exacerbates the risk for adverse cardiovascular outcomes. We present a case of a 58-year-old male with a history of hypertension and IVDU who presented with hypotension, subsequently diagnosed with *Staphylococcus aureus* IE and non-ST-elevation myocardial infarction (NSTEMI) type II. The patient's clinical presentation was marked by severe hypotension and impending shock, prompting an immediate septic workup. Blood cultures revealed the presence of *Staphylococcus aureus* in all four bottles, confirming the diagnosis of IE. Transthoracic echocardiography revealed a large vegetation measuring 1.9x1.7 cm on the mitral valve, further confirming the diagnosis.

In addition to the complications related to IE, the patient's history of cocaine abuse introduced an additional challenge. Cocaine-induced cardiovascular complications, including NSTEMI, pose a unique management dilemma. Furthermore, the patient's concurrent atrial fibrillation and therapeutic anticoagulation necessitated careful consideration of the bleeding risk associated with the existing hemorrhagic stroke. Given the complex nature of the case, a multidisciplinary approach involving cardiology, cardiothoracic surgery, infectious disease, and neurology was employed to optimize the management strategy. Ultimately, due to the high-risk features of the IE and the need to address the hemorrhagic stroke, a decision was made to temporarily halt anticoagulation and proceed with urgent mitral valve replacement surgery.

This case report underscores the challenges encountered in managing patients with multiple comorbidities, including IVDU, cocaine abuse, IE, and cardiovascular and neurological complications. The importance of a comprehensive and collaborative approach to patient care is emphasized, highlighting the need for timely interventions and interdisciplinary coordination.

## Case presentation

A 58-year-old male with a history of hypertension and IVDU presented to the emergency department after feeling unwell and experiencing lightheadedness. On examination, he appeared pale and tachycardic up to 140 beats per minute with a blood pressure of 80/50 mmHg. Septic workup was initiated promptly, including blood cultures and the patient was commenced on broad-spectrum intravenous antibiotics. Initial laboratory results showed leukocytosis and elevated cardiac enzymes as shown in Table 1.

**Table 1:** Laboratory results BNP: Brain natriuretic peptide

Basic labs	Results	Reference range
White blood cells	25.46	3.2-10.6x10^9^/L
Hemoglobin	11.4	14.6-17.8 g/dL
Hematocrit	35.2	40.8-51.9 %
Platelets	144	177-406x10^9^/L
Sodium	138	135-145 mEq/L
Chloride	109	95-111 mEq/L
Blood urea nitrogen	13	7-25 mg/dL
Creatinine	0.90	0.6-1.2 mg/dL
Potassium	4.2	3.5-5.1 mEq//L
Magnesium	2.19	1.7-2.5 mg/dL
BNP	358	<100 Pg/mL
Troponin (Initial)	0.469	<0.03 Ng/mL
Troponin (Peaked)	0.604	<0.03 Ng/mL
Creatinine phosphokinase (CPK)	90	35-230 IU/L

**Figure 1:**
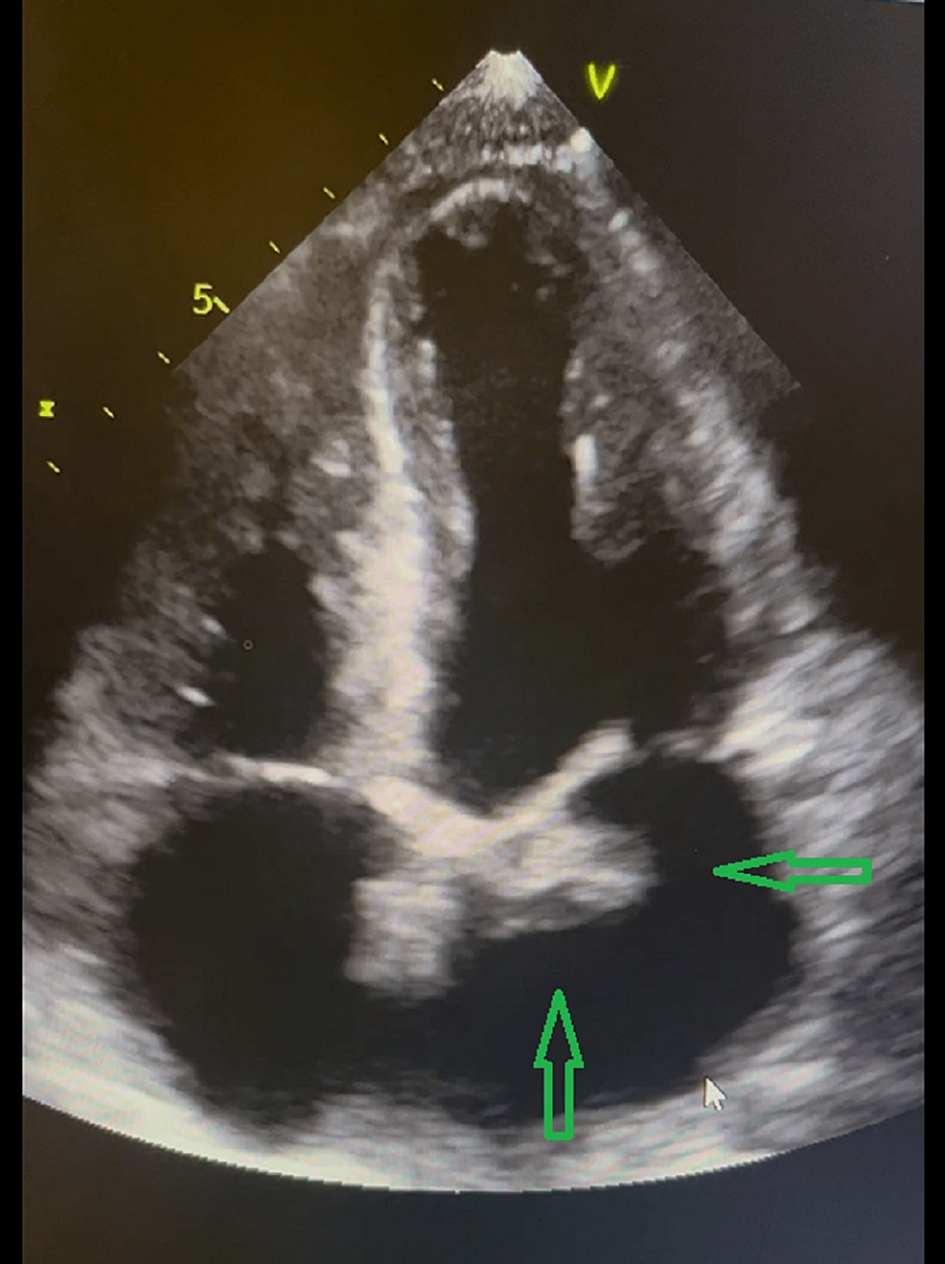
Transthoracic echocardiography four-chamber view showing 1.9x1.7 cm mitral valve vegetation as shown with green arrows

The initial management focused on resuscitation, and the patient showed a favorable response to intravenous fluid administration. However, the blood culture results were concerning, as all four bottles grew *Staphylococcus aureus*. These findings were concerning towards the diagnosis of *Staphylococcus aureus* IE given the previous history of subjective fevers and IVDU. Transthoracic echocardiography was performed, revealing a large vegetation measuring 1.9x1.7 cm on the mitral valve (Figure 1).

**Figure 2:**
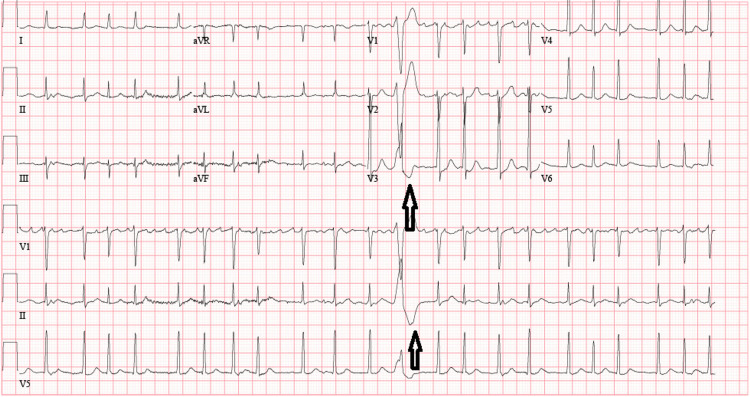
Electrocardiogram (ECG) showing atrial fibrillation with rapid ventricular response along with premature ventricular or aberrantly conducted complex as shown with black arrows

In addition to the IE, the patient's medical history included cocaine and intravenous heroin use. Electrocardiogram findings were consistent with NSTEMI likely type II, in the setting of cocaine use. The patient was already receiving therapeutic anticoagulation due to concurrent atrial fibrillation, further complicating the management strategy (Figure 2).

**Figure 3:**
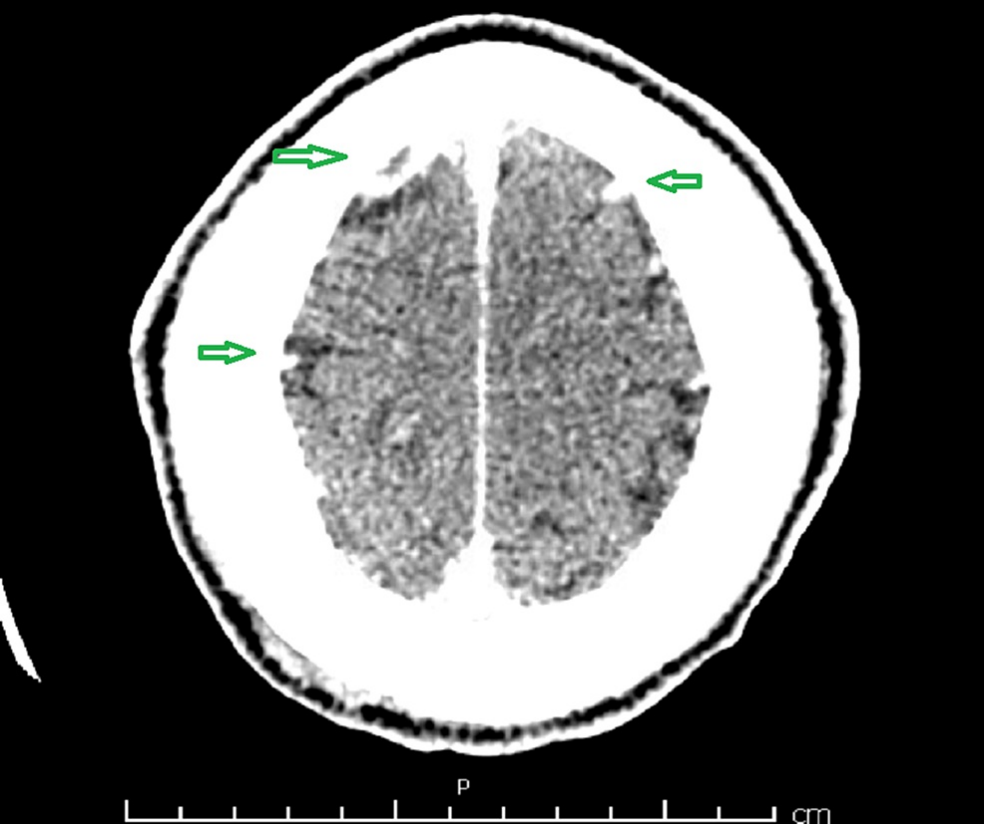
Computed tomography (CT) scan showing areas of cortical hypodensities with hyperdensities representing hemorrhagic stroke bilaterally as shown with green arrows

Further investigations, including neuroimaging, were warranted due to the patient's altered mental status. Imaging studies revealed findings suggestive of a hemorrhagic stroke, necessitating immediate attention from the neurology team as shown in Figure 3. Given the complexity of the case, a multidisciplinary approach involving cardiology, cardiothoracic surgery, infectious disease, and neurology was crucial for formulating an optimal management plan. The infectious disease team recommended targeted antibiotic therapy based on the susceptibility profile of the isolated *Staphylococcus aureus*. Due to the high-risk features of the IE and the need to address the hemorrhagic stroke, the decision was made to temporarily hold anticoagulation and proceed with urgent mitral valve replacement surgery. The patient was transferred to a specialized center with cardiothoracic surgical expertise. The surgical intervention involved mitral valve replacement, aimed at eradicating the source of infection and preventing further embolic events. Postoperatively, the patient's condition gradually improved. Anticoagulation was cautiously reintroduced, balancing the risk of embolism against the risk of hemorrhagic complications. Cardiac rehabilitation was initiated.

This case report highlights the intricate challenges encountered in managing a complex patient with concurrent cocaine-induced NSTEMI, *Staphylococcus aureus* IE and its vascular phenomenon including hemorrhagic stroke and splenic infarct. Successful multidisciplinary collaboration played a pivotal role in guiding the timely intervention for therapeutic decisions, optimizing patient outcomes, and emphasizing the importance of interdisciplinary coordination in such complex clinical scenarios.

## Discussion

The case presented involves a complex patient with concurrent *Staphylococcus aureus *IE, cocaine-induced NSTEMI, and a hemorrhagic stroke. The management of such complex scenarios requires a comprehensive approach that addresses the specific aspects of each condition while considering their interplay [2].

One crucial aspect in the management of IE is the choice of specific antibiotic therapy. *Staphylococcus aureus* is a common pathogen associated with IE, and prompt initiation of appropriate antibiotics is vital for successful treatment [3]. The infectious disease team plays a central role in guiding targeted antibiotic therapy based on the susceptibility profile of the isolated *Staphylococcus aureus* [3]. The selection of antibiotics should consider their efficacy against *Staphylococcus aureus*, including methicillin-resistant strains if present, and their safety profile in the context of the patient's comorbidities and potential drug interactions [4].

Timely surgical intervention is another critical factor in managing complex scenarios involving IE with large vegetation [5]. In this case, the patient presented with large vegetation on the mitral valve, indicating a high risk of embolism. Urgent mitral valve replacement surgery was performed to eradicate the source of infection and prevent further embolic events. Cardiothoracic surgery plays a pivotal role in addressing the primary source of infection and restoring cardiac function. The timing of surgery is a crucial decision, balancing the urgency of the surgical intervention with the patient's overall clinical stability and the risks associated with the procedure [5]. Close collaboration between the cardiology and cardiothoracic surgery teams is essential to determine the optimal timing for surgical intervention and ensure a well-coordinated approach.

Considering the patient's history of intravenous drug use, the management of cocaine-induced NSTEMI presents unique challenges. Cocaine use is associated with various cardiovascular complications, including myocardial infarction [6]. In this case, the electrocardiogram findings were consistent with NSTEMI type II, likely attributed to cocaine use. The management of cocaine-induced NSTEMI involves a multifaceted approach. Initial management focuses on supportive measures, such as adequate oxygenation, pain control, and addressing any hemodynamic instability. Additionally, risk factor modification, including cessation of cocaine use and addressing underlying comorbidities such as hypertension, is crucial. Balancing the use of anticoagulation in the setting of concurrent atrial fibrillation, cocaine-induced platelet activation, and the risk of bleeding due to hemorrhagic stroke requires careful consideration [7]. A thorough assessment of the risks and benefits, in collaboration of the cardiology and neurology teams, is necessary to determine the appropriate approach to anticoagulation in this complex scenario. The decision regarding anticoagulation in patients with both atrial fibrillation and a recent hemorrhagic stroke is challenging. Anticoagulation is typically indicated in atrial fibrillation to reduce the risk of thromboembolic events [8]. However, in the presence of a hemorrhagic stroke, there is a heightened risk of recurrent bleeding. The decision to initiate or withhold anticoagulation must carefully weigh the risk of embolism from atrial fibrillation against the risk of recurrent bleeding from the recent stroke [9].

In this case, the multidisciplinary team made the decision to temporarily hold anticoagulation in the immediate postoperative period following mitral valve replacement. This approach aimed to minimize the risk of bleeding complications related to anticoagulant use during the critical healing phase after surgery [10]. The reintroduction of anticoagulation was cautiously performed after considering the patient's clinical condition, the resolution of the hemorrhagic stroke, and the risk-benefit ratio. Close monitoring of the patient's neurological status and imaging studies guided the decision-making process [11].

The approach to anticoagulation in complex scenarios involving concurrent atrial fibrillation, IE, and a recent hemorrhagic stroke should be individualized and based on a thorough assessment of the risks and benefits. Factors to consider include the patient's overall clinical stability, the extent and severity of the hemorrhagic stroke, the risk of recurrent embolism from atrial fibrillation, and the potential for bleeding complications with anticoagulation. It is important to note that the management decisions regarding specific antibiotic therapy, timely surgical intervention, and anticoagulation in these complex scenarios are based on the available evidence and clinical judgment [12]. The approach may vary depending on the patient's individual characteristics, institutional protocols, and the expertise of the multidisciplinary team involved in the case.

## Conclusions

In complex scenarios involving concurrent infective endocarditis, cocaine-induced non-ST segment elevated myocardial infarction, and a hemorrhagic stroke, the management decisions regarding specific antibiotic therapy, timely surgical intervention, and anticoagulation require a comprehensive and multidisciplinary approach. The selection of specific antibiotic therapy should be guided by the susceptibility profile of the isolated pathogen, considering efficacy and safety. Timely surgical intervention, such as mitral valve replacement, is essential to eradicate the source of infection and prevent further embolic events. The decision regarding anticoagulation must carefully balance the risks of embolism from atrial fibrillation and the risk of recurrent bleeding from the recent hemorrhagic stroke. The approach to anticoagulation should be individualized and based on a thorough assessment of the patient's clinical condition, the extent and severity of the hemorrhagic stroke, and the risk-benefit ratio. Multidisciplinary collaboration, evidence-based decision-making, and a patient-centered approach are crucial for optimizing outcomes in these complex clinical scenarios.

## References

[1] Murdoch DR, Corey GR, Hoen B (2009). Clinical presentation, etiology, and outcome of infective endocarditis in the 21st century: the International Collaboration on Endocarditis-Prospective Cohort Study. Arch Intern Med.

[2] Wei XB, Huang JL, Liu YH (2020). Incidence, risk factors and subsequent prognostic impact of new-onset atrial fibrillation in infective endocarditis. Circ J.

[3] Hidalgo-Tenorio C, Gálvez J, Martínez-Marcos FJ (2020). Clinical and prognostic differences between methicillin-resistant and methicillin-susceptible Staphylococcus aureus infective endocarditis. BMC Infect Dis.

[4] Lefèvre B, Hoen B, Goehringer F (2021). Antistaphylococcal penicillins vs. cefazolin in the treatment of methicillin-susceptible Staphylococcus aureus infective endocarditis: a quasi-experimental monocentre study. Eur J Clin Microbiol Infect Dis.

[5] Prendergast BD, Tornos P (2010). Surgery for infective endocarditis: who and when?. Circulation.

[6] Chibungu A, Gundareddy V, Wright SM, Nwabuo C, Bollampally P, Landis R, Eid SM (2016). Management of cocaine-induced myocardial infarction: 4-year experience at an urban medical center. South Med J.

[7] Hart RG, Foster JW, Luther MF, Kanter MC (1990). Stroke in infective endocarditis. Stroke.

[8] Preston AH, Williams S, Archer J (2016). A review of the role of anticoagulation for patients with infective endocarditis and embolic stroke. Clin Case Rep.

[9] Rasmussen RV (2011). Anticoagulation in patients with stroke with infective endocarditis is safe. Stroke.

[10] Hart RG, Kagan-Hallet K, Joerns SE (1987). Mechanisms of intracranial hemorrhage in infective endocarditis. Stroke.

[11] Sotero FD, Rosário M, Fonseca AC, Ferro JM (2019). Neurological complications of infective endocarditis. Curr Neurol Neurosci Rep.

[12] Jussli-Melchers J, Salem MA, Schoettler J (2022). Mid- and long-term surgical outcomes due to infective endocarditis in elderly patients: a retrospective cohort study. J Clin Med.

